# Lesion Mapping the Four-Factor Structure of Emotional Intelligence

**DOI:** 10.3389/fnhum.2015.00649

**Published:** 2015-12-10

**Authors:** Joachim T. Operskalski, Erick J. Paul, Roberto Colom, Aron K. Barbey, Jordan Grafman

**Affiliations:** ^1^Decision Neuroscience Laboratory, University of IllinoisUrbana, IL, USA; ^2^Beckman Institute for Advanced Science and Technology, University of IllinoisUrbana, IL, USA; ^3^Neuroscience Program, University of IllinoisUrbana, IL, USA; ^4^Department of Biological and Health Psychology, Universidad Autónoma de MadridMadrid, Spain; ^5^Department of Internal Medicine, University of IllinoisUrbana, IL, USA; ^6^Department of Psychology, University of IllinoisChampaign, IL, USA; ^7^Department of Speech and Hearing Science, University of IllinoisChampaign, IL, USA; ^8^Cognitive Neuroscience Laboratory, Brain Injury Research Program, Rehabilitation Institute of ChicagoChicago, IL, USA

**Keywords:** emotional intelligence, MSCEIT, social cognition, voxel-based lesion-symptom mapping, traumatic brain injury

## Abstract

*Emotional intelligence (EI)* refers to an individual’s ability to process and respond to emotions, including recognizing the expression of emotions in others, using emotions to enhance thought and decision making, and regulating emotions to drive effective behaviors. Despite their importance for goal-directed social behavior, little is known about the neural mechanisms underlying specific facets of EI. Here, we report findings from a study investigating the neural bases of these specific components for EI in a sample of 130 combat veterans with penetrating traumatic brain injury. We examined the neural mechanisms underlying experiential (perceiving and using emotional information) and strategic (understanding and managing emotions) facets of EI. Factor scores were submitted to voxel-based lesion symptom mapping to elucidate their neural substrates. The results indicate that two facets of EI (perceiving and managing emotions) engage common and distinctive neural systems, with shared dependence on the social knowledge network, and selective engagement of the orbitofrontal and parietal cortex for strategic aspects of emotional information processing. The observed pattern of findings suggests that sub-facets of experiential and strategic EI can be characterized as separable but related processes that depend upon a core network of brain structures within frontal, temporal and parietal cortex.

## Introduction

*Emotional intelligence* (EI) refers to a set of competencies that enable people to engage in information processing about emotions and to use this information to guide thought and behavior (Mayer et al., 2008b). Evidence indicates that EI engages a distributed network of frontal and parietal structures that also enables goal-directed behavior more generally (Barbey et al., 2014a). Historically, neuropsychological research has emphasized the functional contributions of the prefrontal cortex (PFC), showing that networks incorporating the PFC are central to goal-directed behavioral control (Dehaene et al., 1998; Miller and Cohen, 2001). Indeed, executive and social functions are frequently disrupted by traumatic brain injuries affecting those networks, posing fundamental difficulties for successful rehabilitation (Barbey and Grafman, 2011; Barbey et al., 2011, 2012a,b, 2013, 2014a,c; Koenigs et al., 2011, 2009). Questions remain about the relationships between emotional processes, general intelligence, and their neural correlates that are the subject of ongoing research and scientific exchange. In particular, questions about the unitary nature of EI and whether specific facets of EI share common or distinct neural mechanisms from one another are interrelated questions of central importance to intelligence researchers (Mayer et al., 2001; Bar-On et al., 2003; Matthews et al., 2004). The goals of this research program are twofold: to characterize the fundamental nature of human intelligence by studying how impairments arise, and to use that knowledge to improve the state of treatment for those with cognitive and emotional or social impairments.

In prior research, a network of neural correlates has been linked to social and emotional processing using a range of experimental materials such as interpersonal scenarios, cartoons, jokes, sarcasm, faux pas, and moral decision-making tasks. Analysis of the specialized contributions of different brain regions has suggested, for example, that the orbitofrontal cortex plays a key role in the representation of mental states; the right posterior superior temporal sulcus supports the perception of biological motion or agency, facilitating the interpretation of purposeful movements; the left temporal pole is involved in storing relevant social knowledge, which contributes to the contextual understanding of others’ social interactions; and orbitofrontal-amygdala regions are involved in emotional aspects of social processing (Frith and Frith, 2003; Sabbagh, 2004; Moll et al., 2011). Available evidence converges on the finding that tests of empathy or other social and emotional processing engage this social knowledge network, but several questions remain open. A primarily right-hemispheric network for emotional empathy has been suggested on the basis of imaging evidence and confirmatory brain lesion studies (Leigh et al., 2013; Hillis, 2014), but other findings posit key contributions to “mentalizing” from the analogous left-hemisphere brain regions and bilateral networks as well (Frith and Frith, 2003). Although studies consistently link emotion processing and social cognition to the prefrontal, temporal and insular lobes, the nature of that functional mapping is complex (Takeuchi et al., 2011, 2013a,b; Tan et al., 2014).

Previously, we reported lesion evidence mapping EI to parts of a social cognition network in the brain: a distributed system spanning left frontal, temporal and parietal regions (Barbey et al., 2014a). Removing the variance in the model shared between EI and other psychological constructs – variables including general intelligence (verbal comprehension, perceptual organization, working memory, and processing speed) and such personality traits as openness, extraversion and neuroticism – revealed that EI was diminished with lesions in the right orbitofrontal cortex and left parietal cortex. The results suggest that EI relies to some extent on brain structures that support cognitive functions in the classic psychometric intelligence construct, but it also involves capabilities specific to social and emotional information. Here, we explore the relationship between brain structure and EI further, probing the same cohort of brain lesion patients for insight concerning the nature of their deficits in EI factors. One model of EI characterizes it as a set of distinct, yet correlated, competencies (Mayer et al., 2004); it remains to be seen whether the previously reported impairments in EI are behaviorally similar across participants irrespective of the details of their injuries, or are driven by different competencies depending on the nature and extent of a particular person’s damage to social cognition or emotion-experiencing networks. For example, other cognitive constructs (including general intelligence and language processing) have been mapped to networks in the brain featuring a domain-general “core” network, with task-specific and specialized “peripheral” nodes selectively engaged by only some tasks or cognitive sub-functions (Bassett et al., 2013; Fedorenko and Thompson-Schill, 2014). Motivated by the psychometric features of the factor structure of EI (chiefly, that there is both shared and specific variance), we hypothesize a core-periphery mapping of function to structure among the participants in our sample with impaired EI: a domain-general “core” conferring deficits in aspects of EI shared among the four factors, and a task-specific “periphery” uniquely supporting the individual facets.

Social and emotional intelligence are not homogeneous constructs, and are instead comprised of specific competencies for goal-directed social behavior (Mayer et al., 2008a,b). Indeed, one influential model posits four conceptually distinguishable sociable factors that together describe the full capacity to process and act on emotions (each of the four falling under one of two larger domains that assist in conceptualizing them in the context of personal emotional experience). The ability to recognize the expression of emotions of others (perceiving emotions), along with the ability to use emotions in generating thoughts and actions (using emotions), falls into the category of *experiential emotional intelligence.* The abilities to grasp the nuances of different emotions and how they relate to one another (understanding emotion) and to regulate one’s own emotions to drive effective behaviors (managing emotions) are considered *strategic emotional intelligence* (Mayer et al., 2008b; Barbey et al., 2014a).

According to this framework, the neural mechanisms underlying EI may reflect the specific competencies of emotional processing that are engaged (Barbey et al., 2014a; Krueger et al., 2009). Cognitive reappraisal, for example, is a strategy for reinterpreting negative events and the emotions they elicit in an attempt to manage affect (Gross, 2002). Reappraising affective states increases dorsal prefrontal activation while inhibiting amygdala and ventromedial PFC (vmPFC) in the context of negative emotion, and it activates amygdala and vmPFC in the context of positive emotions (Ochsner et al., 2002, 2004). The ability to manipulate abstract emotional information in the service of managing future affective states should engage similar prefrontal regions to those involved in real-time affective monitoring and regulation, in addition to a broader information processing network in the brain. We hypothesized that the factors of EI are each supported by a network in frontal, temporal and parietal cortex; specialized nodes of these networks for emotional value or behavioral control should form the “periphery” of the network specific to one or few aspects, while more domain-general nodes that support multiple functions should form the “core” of EI in the brain (e.g., the temporal lobe and insula, that widely support knowledge of others’ intentions and one’s own internal states, respectively).

Although emotional intelligence has been validated as a reliable behavioral construct and has captured public attention as an important addendum to classic models of intelligence, it remains unclear how best to characterize it: different models suggest a personality-like model of trait factors, a set of correlated skills comprising a separate intelligence, and even just the application of traditional factors of intelligence to contexts with emotional arousal or emotional information (Mayer et al., 1999, 2001; Roberts et al., 2001). To more fully understand the nature of EI in the brain, it is necessary to compare results from neuroimaging and neuropsychological studies using measures from each of the contrasting models.

We thus investigated the neural foundations of experiential and strategic EI in a cohort of combat veterans with focal brain injuries (*n* = 130), examining performance on a battery of tests designed to measure skills in each of the factors of EI. By mapping deficits in each facet of EI separately, we tested (i) the selectivity of cortical networks for specific facets of experiential and strategic EI and (ii) the degree to which these systems engage common neural networks. Prior work on the current sample revealed several preliminary findings. Self-reported ability to empathize with others was seen to rely on ventrolateral PFC, insular cortex and the posterior temporal lobes (Driscoll et al., 2012). Affective theory of mind, or the ability to predict and perceive the emotional states of others, was observed among participants with damage to the left ventromedial PFC when comparing their performance to that of subjects with damage in the other hemisphere or elsewhere in the brain (Leopold et al., 2012). Focusing on the experiential and strategic EI of participants with damage to different subregions of interest within the PFC revealed a dissociation of functions along the ventromedial–dorsolateral axis (Krueger et al., 2009), but it remains to be seen how those subregions contribute to the facets of EI as part of functionally defined networks in the brain in the entire sample, rather than by using anatomical landmarks to characterize the smaller subsample of participants with focal damage to regions of interest. Voxel-based lesion-symptom mapping (VLSM) of deficits in the composite EI scores from the Mayer–Salovey–Caruso Emotional Intelligence Test (MSCEIT) revealed a network engaging the left frontal and temporal lobes, in addition to white matter tracts connecting the frontal lobes with the temporal and parietal lobes (Barbey et al., 2014a). VLSM of performance on a task requiring visual facial emotion recognition revealed a frontal–temporal–insular network for perceiving emotions (Dal Monte et al., 2012), although the other facets of EI have yet to be explored at a similar voxel-level resolution. The current analysis is thus a more fine-grained addition to the original MSCEIT-based study, undertaken with the goal of understanding how the different components of a social knowledge network contribute to the behaviorally distinguishable aspects of EI. Furthermore, we intended to explore whether the divergence in findings between those from the current sample and those from other studies probing emotion processing could be explained by lesion-mapping the sub-facets separately from one another. Toward this end, we obtained latent scores on each facet of EI for every participant, which we then used as the basis of a VLSM analysis.

## Materials and Methods

### Participant Data

The Vietnam Head Injury Study (VHIS) was set up by William F. Caveness, chief of the Laboratory of Experimental Neurology at the National Institute of Neurological and Communicative Disorders and Stroke. Caveness assembled the VHIS patient registry, which gathered information from medical records on 1,221 Vietnam veterans who sustained a traumatic brain injury (TBI) between 1967 and 1970 (Raymont et al., 2011). The war in Vietnam was the first to allow early treatment by full surgical teams close to areas of combat operations, due to large-scale helicopter evacuation. Most patients were treated within hours of their battlefield injuries, dramatically increasing survival rates (Rish et al., 1983). In addition, the low-velocity, penetrating fragment wounds that were typically sustained (in combination with neurosurgical debridement) resulted in relatively focal lesions. Participants in this analysis were drawn from Phase 3 of the VHIS registry; all participants are American male veterans who suffered brain damage from penetrating head injuries in the Vietnam War (*n* = 130). This study was approved by the National Naval Medical Center and National Institute for Neurological Disorders and Stroke Institutional Review Boards and, in accordance with stated guidelines and the Declaration of Helsinki, all subjects read and signed informed consent documents. Phase 3 testing occurred between April 2003 and November 2006. Sample means for age, lesion size, pre-injury intelligence, and education of the VHIS subjects included in this analysis are reported in **Table 1**.

**Table 1:** Demographic data.

	Age	Lesion volume	Pre-injury AFQT	Education
Sample mean	59 (3.3)	3.0 (3.3)	65.4 (23.0)	15.0 (2.5)

*Sample means are displayed, with standard deviations in parentheses. Lesion volume is presented as percentage of total brain volume. Armed Forces Qualification Test (AFQT) is an intelligence test score presented as the mean percentile ranking. Education is listed in years of formal schooling*.

### Lesion Analysis

Computed tomography (CT) data were acquired during the Phase 3 testing period. CT is the only non-invasive brain imaging method that is both safe for individuals with ferrous artifacts in their bodies (unlike magnetic resonance) and allows imaging beneath the cortical surface (unlike optical imaging); the spatial resolution of lesion borders by CT is comparable to that for MRI. Axial images were acquired without contrast in helical mode on a GE Electric Medical Systems Light Speed Plus CT scanner at the Bethesda Naval Hospital. Structural neuroimaging data were reconstructed with an in-plane voxel size of 0.4 mm × 0.4 mm, an overlapping slice thickness of 2.5 mm, and a 1 mm slice interval. Lesion location and volume from CT images were determined using the interactive Analysis of Brain Lesions (ABLe) software implemented in MEDx, version 3.44 (Medical Numerics). Lesion volume was calculated by manually tracing the lesion in all relevant slices of the CT image in native space, and then summing the trace areas and multiplying by slice thickness. Manual tracing was performed by a trained psychiatrist with clinical experience in reading CT scans. The lesion tracing was then reviewed by an observer who was blind to the results of the clinical evaluation and neuropsychological testing, enabling a consensus decision to be reached regarding the limits of each lesion. The CT image of each individual’s brain was normalized to a CT template brain image in Montreal Neurological Institute (MNI) space. The spatial normalization was performed with the automated image registration (AIR) algorithm, using a 12-parameter affine fit. Note that both the patient’s brain and the CT template brain are first skull-stripped to maximize the efficacy of the AIR registration from native space to MNI space. In addition, voxels inside the traced lesion were not included in the spatial normalization procedure. For each subject, a lesion mask image in MNI space was saved for VLSM.

### Neuropsychological Tests

Emotional intelligence scores were obtained from the MSCEIT, a standard test of EI (Mayer et al., 2008a). The MSCEIT as a 141-item scale focuses on emotion-related competencies that can be assessed through performance-based standardized norms. Responses on the MSCEIT were scored with respect to their degree of correctness, as determined by their correspondence with the answers provided by a normative sample of the general population. Besides the Full-Scale Emotional Intelligence, the MSCEIT yields two area scores each combining two branch scores: (i) Experiential EI is measured by tasks that require the perception of emotions (i.e., to perceive and identify emotions both in oneself and in others; for example, the ability to accurately read facial expressions) and the use of emotions (i.e., to harness emotions to facilitate thinking; for example, anticipating another person’s emotional reaction and using that knowledge to modify one’s own behavior); and (ii) Strategic EI is measured by tasks that require understanding emotions (i.e., to realize the causes of emotions; for example, understanding the relationship between sadness and loss) and managing emotions (i.e., to apply effective strategies that use emotions to achieve a goal; for example, conscious regulation of emotions both in oneself and in others). A more detailed discussion of the psychometric properties of the MSCEIT can be found in the MSCEIT user’s manual (Mayer et al., 2002; see also Mayer et al., 2004, 2008a,b).

### Factor Analysis and Computed Scores

The eight subtests of the MSCEIT battery were submitted to a factor analysis (Principal Axis Factoring followed by an oblique Promax Rotation) and four correlated factors were obtained (**Table 2**). The KMO value of the obtained factor solution was 0.70 and the Bartlett test was significant (Chi-square = 195, DF = 28, *p* = 0.000). **Table 2** shows the factor loadings for the eight subtests on the four factors. Note that subtests theoretically belonging to the same EI facet (perceiving emotions: faces and pictures; using emotions: sensations and facilitation; understanding emotions: blends and changes; and managing emotions: emotions management and social management) show remarkable loadings on their respective factor. Correlations among the obtained factors are of moderate magnitude, ranging from 0.26 to 0.52. Finally, four latent scores were obtained applying the regression method provided with the software (SPSS). The use of factor scores in subsequent lesion-symptom mapping instead of raw subtest scores enables the isolation of key competencies of interest, removing task-specificity.

**Table 2:** Factor matrix and correlations among the obtained factors.

MSCEIT	F1	F2	F3	F4
**Perceiving emotions**
Faces	0.04	0.34	**0.68**	0.11
Pictures	0.24	0.35	**0.56**	0.31
**Using emotions**
Sensations	0.31	0.58	0.39	**0.55**
Facilitation	0.14	0.25	0.16	**0.64**
**Understanding emotions**
Blends	**0.61**	0.31	0.21	0.25
Changes	**0.82**	0.29	0.09	0.15
**Managing emotions**
Emotion management	0.29	**0.77**	0.40	0.32
Social management	0.50	**0.68**	0.53	0.39
F2	0.45			
F3	0.26	0.60		
F4	0.33	0.52	0.37	

*Values in bold represent the loadings for each subtest on the corresponding latent variable from the four-factor structure*.

### Lesion-Symptom Mapping

The computed latent scores for each subject were correlated with regional brain damage determined by VLSM (Bates et al., 2003). The classical region-of-interest (ROI) approach to studying the effects of focal brain damage is performed by comparing a group of study participants with similar brain damage profiles (similar in size and location) to participants in a matched control group with either no brain damage or only damage to regions outside the region of interest. VLSM, by contrast, leverages the diversity of brain damage profiles in a large sample of patients to increase statistical power while still controlling for the effects of brain injury not accounted for by focal tissue damage. More specifically, an overlap map of the entire sample’s lesions is created and then masked to include only the voxels where a large enough number of subjects sustained damage. Convention for the minimum number of subjects with a lesion in a voxel to include it in the analysis is typically 3 or 4 (Barbey et al., 2012a; Gläscher et al., 2012); we conducted both analyses and report the results using three subjects here in order to obtain a broader spread of lesion sampling. Supplementary Figures S4 and S5 display the results using a four subject minimum, in which the visually discernable structures from the three-subject results are all represented, although with fewer voxels meeting criterion for significance. After creating the lesion overlap mask for analysis, “lesion” and “control” groups are created iteratively within the entire sample at each voxel in the mask, such that the scores of a psychometric test can be compared between the groups with damage in the voxel of interest and those with no damage in that particular location; the brain regions outside the voxel of interest effectively become the “control” regions to account for the general effects of brain injury irrespective of injury location. We used an independent samples *t*-test to compare the groups at each voxel, and conducted a False Discovery Rate correction of *q* < 0.05 to limit the proportion of false positives within the total number of tests found to be statistically significant. We conducted our analyses using the statistics toolbox in MATLAB (Version R2014b) and visualized the results in MNI space using FSL (Version 5.0) (Jenkinson et al., 2012).

## Results

### Descriptive Results and Correlation Matrix

**Table 3** shows the descriptive results for the eight subtests of the MSCEIT (namely, faces, pictures, sensations, facilitation, blends, changes, emotion management, and social management), along with the correlation matrix. Supplementary Table 1 shows the correlations between each of the four factors and lesion size.

**Table 3:** Descriptive results and correlation matrix for the eight subtests of the Mayer–Salovey–Caruso Emotional Intelligence Test (MSCEIT).

		Mean	*SD*	1	2	3	4	5	6	7	8
(1)	Faces	108	38	–	0.35^∗∗^	0.18^∗^	0.02	0.06	–0.05	0.23^∗∗^	0.30^∗∗^
(2)	Pictures	97	17		–	0.29^∗∗^	0.14	0.20^∗^	0.11	0.22^∗^	0.31^∗∗^
(3)	Sensations	94	14			–	0.32^∗∗^	0.16	0.22^∗^	0.43^∗∗^	0.40^∗∗^
(4)	Facilitation	106	18				–	0.15	0.03	0.13	0.20^∗^
(5)	Blends	89	11					–	0.49^∗∗^	0.21^∗^	0.33^∗∗^
(6)	Changes	96	13						–	0.15	0.35^∗∗^
(7)	Emotion management	93	11							–	0.50^∗∗^
(8)	Social management	90	11								–

*^∗^*p* < 0.05, ^∗∗^*p* < 0.01*.

### Voxel-Based Lesion-Symptom Mapping

Voxel-based lesion-symptom mapping revealed selective deficits in specific facets for experiential and strategic EI, identifying a core network of brain structures that underlie the perception and management of emotional information.

### Perceiving Emotions

**FIGURE 1:**
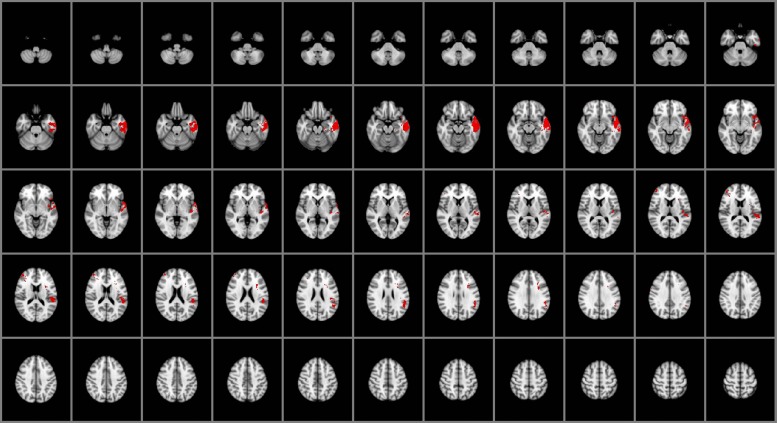
**Voxel-based lesion-symptom mapping (VLSM) of perceiving emotions (*n* = 130)**. The statistical brain map of VLSM results in Montreal Neurological Institute (MNI) space is thresholded at *q* < 0.05 (using a false discovery rate correction for multiple comparisons). In each axial slice, the right hemisphere is on the reader’s left.

Impairments in the ability to perceive emotional information (**Figure 1**) were associated with selective damage to the right lateral frontal pole and middle frontal gyrus (BA 45, 46), left temporal pole (BA 38), fusiform gyrus (BA 37), inferior temporal cortex (BA 20 and 21), posterior superior temporal gyrus (BA 41, 42 and 22), insular cortex, and the parietal operculum (BA 40). Focal damage associated with impaired experiential EI was not limited to cortical gray matter structures, however. Damage was also observed within the left superior longitudinal fasciculus and the left uncinate fasciculus.

### Managing Emotions

**FIGURE 2:**
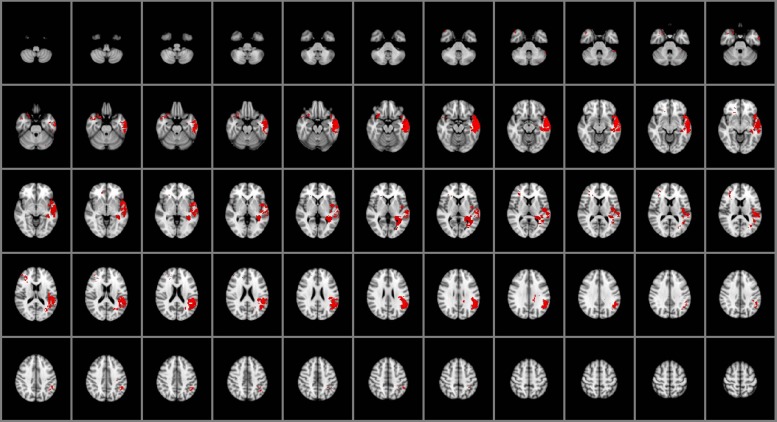
**Voxel-based lesion-symptom mapping of managing emotions (*n* = 130)**. The statistical brain map of VLSM results in MNI space is thresholded at *q* < 0.05 (using a false discovery rate correction for multiple comparisons). In each axial slice, the right hemisphere is on the reader’s left.

As illustrated in **Figure 2**, impairments in the capacity to manage emotions were associated with damage to the right posterior orbitofrontal cortex (BA 10), bilateral temporal poles (BA 38), and left middle and superior temporal gyri (BA 21, 22, 41, 42), left insula and left posterior hippocampus. Emotion management was also selectively associated with damage to left ventral parietal regions, including the angular gyrus (BA 39), supramarginal gyrus and parietal operculum (BA 40). White matter involvement included the left superior longitudinal fasciculus and the adjacent, posterior aspects of the left inferior longitudinal fasciculus.

### Common and Distinctive Regions for Individual Facets of EI

**FIGURE 3:**
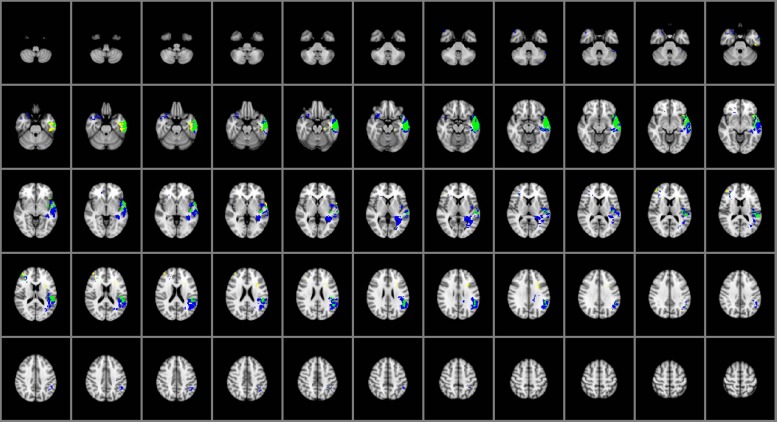
**Brain regions common and distinctive to perceiving and managing emotion (*n* = 130)**. Yellow: perceiving. Blue: managing. Green: common to both. In each axial slice, the right hemisphere is on the reader’s left. Jaccard Index = 0.50; η^2^ = 0.77

The overlap map in **Figure 3** displays the brain regions common and distinctive to the different domains of EI. Damage within the left temporal pole, left middle, and superior temporal gyri and left insular cortex predicted impairments in both perceiving and managing emotions. Perceiving emotion alone was impaired among participants with damage to the left fusiform gyrus, medial aspects of the left ventral temporal cortex and right middle frontal gyrus; only managing emotions was impaired in participants with damage to the left angular and supramarginal gyri, left posterior temporal lobe, the left longitudinal fasciculi, the right temporal pole, and right caudal orbitofrontal cortex. Using emotion (which falls under the experiential domain) and understanding emotion (which falls under the strategic domain) were not sensitive to brain damage at the voxel-level in this sample of brain lesion patients.

## Discussion

Prior efforts to investigate the EI of participants in the VHIS Registry revealed that composite scores of EIQ from the MSCEIT engaged the core network for social cognition, including left posterior temporal cortex, left temporo-parietal junction, left orbitofrontal cortex, and key white matter tracts connecting frontal, temporal, and parietal regions (Barbey et al., 2014a). Measures of practical problem solving in social contexts revealed an impairment among those with damage to the social cognition network in addition to bilateral orbitofrontal cortex (Barbey et al., 2014b), whereas affective theory of mind was impaired among those with damage to the left ventromedial PFC (Leopold et al., 2012).

In this study, we advance our prior research on the neural mechanisms of general EI by providing an empirical basis for understanding the neural architecture of specific facets of EI. The results indicate that specific facets of experiential and strategic EI (perceiving and managing emotions, respectively) engage common and distinctive neural systems, with shared engagement across the social processing and conceptual knowledge networks, selective engagement of the orbitofrontal and parietal cortices for managing emotions (**Figure 2**), and similarly selective engagement of the medial ventral temporal cortex and lateral PFC for perceiving emotions (**Figure 1**). The observed pattern of findings suggests that the facets of EI can be characterized as separable but related processes supported by a core network of brain structures within frontal, temporal, and parietal cortex.

The core network of regions underlying EI comprises a neural system that has been linked to social and emotional processing (Moll et al., 2005; Zahn et al., 2007, 2009). Analysis of the specialized contributions of regions within the social knowledge network has suggested, for example, that the orbitofrontal cortex plays a key role in the representation of mental states, both for an individual’s own thoughts and beliefs and those of others (Saxe and Kanwisher, 2003; Saxe, 2006), with significant contributions by the temporal and parietal cortex in assessing and simulating the mental states of others (Saxe and Kanwisher, 2003). The anterior ventral temporal lobe in particular is widely implicated in such higher-order functions as integrating social, emotional, and conceptual knowledge in concert with frontal lobe activity and more stimulus-specific information from the primary and secondary sensory cortices (Frith and Frith, 2003). Thus, the network observed in the current analysis engages regions implicated in EI and may reflect key competencies for social information processing. As illustrated in **Figure 3**, the regions implicated in facets of both strategic and experiential EI (left frontal, temporal, and insular cortex) are among those central to social cognition and known to underlie the perception of emotionally relevant information.(Hoffman and Haxby, 2000; Adolphs, 2002; Frith and Frith, 2003; Saxe and Kanwisher, 2003; Saxe, 2006; Reis et al., 2007; Gu et al., 2012).

Some brain regions were only associated with impairments to a single factor of EI, suggesting that they form the task-specific periphery of the networks supporting EI and social cognition. Only the “perceiving emotions” factor was impaired after damage to regions in the ventral temporal lobe, a region that has previously been shown to support categorical conceptual knowledge (Kanwisher et al., 1997; Gauthier et al., 1999) and may support visual recognition of emotional expression (Harry et al., 2013). The “managing emotions” factor selectively engaged the right posterior orbitofrontal cortex, which has been increasingly implicated in decision making and emotional processing (Bechara et al., 2000), as a nexus of integrating executive, social and emotional processes (Kringelbach, 2005). Managing emotions also engaged the posterior hippocampus, implicating relational memory systems in the strategic component of processing emotional information (Eichenbaum et al., 1994). Activity in the hippocampus has been suggested to support the interpretation and prediction of others’ emotional states through the elicitation of autobiographical episodic memory in concert with the typical theory of mind network (Perry et al., 2011). Finally, regions of parietal cortex were also related to impaired managing of emotion, namely the superior parietal regions previously associated with affect-free attention and executive control (Miller and Cohen, 2001), and ventral parietal regions previously associated with theory of mind, episodic memory, and language processing (Cabeza et al., 2012).

Key white matter tracts were among the brain regions in the VLSM results as well. This offers support for the importance of brain networks in EI, as opposed to a modular view of the brain. Both perceiving and managing emotions rely on the left superior longitudinal fasciculus, which connects the frontal pole with the superior parietal lobe and enables the transfer of information across the fronto-parietal network. Damage to adjacent, posterior aspects of the left inferior longitudinal fasciculus was associated with lower performance on emotion management tasks only. This suggests that integration of visual signals from the occipital lobe with the social knowledge representations in the frontal cortex is critical to the ability to manipulate and control emotions in a social context. Rostrally, damage in the left uncinate fasciculus was specific to deficits in the perceiving of emotions, further implicating fronto-temporal integration and the social knowledge network as major supporters of the ability to recognize emotions in others. Integrated signaling between the frontal, parietal, temporal, and occipital lobes occurs obligatorily and automatically during any successful human communication (Saxe and Kanwisher, 2003). The fasciculi and cortical white matter association tracts are important for such integrated signaling between regions, explaining why their damage predicts risk of impaired EI in multiple domains.

Prior lesion study findings using ROI methods (Bar-On et al., 2003) appear to be at odds with the results of the voxel-wise approach reported here, but it is possible to reconcile the difference by considering them together and in the context of their respective theoretical motivations. Viewing EI as a domain including both personality traits and a set of quantitative competencies explains why self-report and trait-based measures of EI (e.g., EQ-i) are sensitive to categorical traits like the ability to experience a particular emotion or the perception of being sensitive and skilled in interpersonal communication. The inability to represent emotion as an abstract concept and use it in decision-making is an example of the impairments that can be revealed by trait-based or mixed models of EI, and this inability has been observed after damage to a very specific emotion-representation network in the brain (Bechara et al., 1999). Skill-based measures of EI (e.g., MSCEIT, as used in the current study) are sensitive instead to quantitative differences in the ability to perceive increasingly nuanced differences in emotions of others, or the ability to successfully navigate social scenarios with increasingly complex or ambivalent emotions, for example. As with measures of psychometric *g*, quantitative differences in a wide range of emotion processing steps reveal a more distributed network in the brain, including substrates traditionally characterized as supporting pure affective experience, memory retrieval for contextual details, and such executive functions as set shifting and cognitive updating.

We note that prior analysis of the psychometric structure of EI in the current sample found a significant correlation between general EI and verbal comprehension, processing speed, and conscientiousness (Barbey et al., 2014a). This is also the case for the factors of EI, suggesting that these factors of EI are intertwined with these other aspects of cognition and personality. Nevertheless, it is also highly likely that our study materials engage fundamental processes other than the target EI processes of interest (e.g., language proficiency to read and respond to written vignettes). The methods reported here do not offer sufficient evidence for a mediating link between EI and any correlated cognitive ability or personality trait, nor do they offer evidence for particular neural structures being necessary and sufficient for the EI factors we measured. We interpret our findings as evidence for a relationship between local structural brain integrity and the ability to process emotional and social information. From a brain network perspective, the brain regions identified in the current analysis function as part of larger networks that support multiple and related functions – functions of which some may be critical for EI while others may engage similar neural structures without making genuine contributions to emotionally intelligent behavior.

### Implications, Limitations, and Conclusion

From a clinical perspective, understanding impairments in EI in patients with brain damage may facilitate the design of appropriate assessment tools and rehabilitation strategies, with potential improvement in patients’ cognitive abilities (e.g., problem solving, self-expression, adaptability) and daily living. The reported findings identify markers that may be targeted in clinical investigations to assess the functioning of the EI network, particularly measures of experiential and strategic information processing. Some brain structures are engaged by several facets of EI, whereas other regions are involved in one facet but not others (**Figures 1–3**). These findings support predictions about the nature and significance of social and emotional impairments that may result from damage to specific brain regions.

Indeed, many neurological disorders and mental illnesses are characterized by deficits in emotional and cognitive behaviors, including epilepsy, Alzheimer’s disease, autism, and schizophrenia. Outstanding questions concerning these and many other debilitating conditions center on advancing our knowledge of how emotional and cognitive processes interact in both normal and abnormal circumstances. Understanding the neural mechanisms underlying these conditions will ultimately require a broader assessment that examines the functional organization of emotional and cognitive systems, and their interactive role in high-level processes. The reported findings contribute to this research program by elucidating the neural architecture of emotional processes, suggesting that core facets of EI emerge from a distributed network of brain regions that support specific competencies for human intelligence.

Finally, the utility and shortcomings of large-sample brain lesion-symptom mapping should not go unnoticed. The remarkable statistical power of being able to non-invasively image the brain activities of as many healthy participants as allowed by funding constraints has resulted in a rich literature on the neural correlates of many social and emotional cognitive processes. Functional neuroimaging alone can not discriminate between the necessary and redundant components of any particular pattern of brain activity, however, and the slow nature of the BOLD response makes decomposing a time series of fMRI data into single events within a sequence challenging. Carefully controlled brain damage studies are useful to complement what gaps may be present in the functional imaging literature; chiefly, the comparative loss and preservation of function after damage to different brain regions among well-matched study groups provides information about the necessity of nodes within distributed brain networks. This lesion analysis – and the ones that preceded it – builds on prior neuroimaging research. This analysis in particular reveals that not all EI facets are equally sensitive to brain damage in this sample.

The limitation of lesion research to be taken into account alongside its strengths is simply that brain lesion studies are poised to identify the effects of damage to regions affected in a particular sample, but remain silent about the contributions of regions that remain intact in the whole sample. VLSM in particular is only able to reveal structure-function mappings in regions meeting the threshold for inclusion in the lesion overlap map. In Supplementary Figures S1–S3, we display the lesion overlap map for this sample, in addition to voxel-wise Cohen’s-*d* maps for each of the facet-specific lesion-symptom mappings that resulted in statistically significant findings; note that subcortical structures are not well represented in the current study’s lesion overlap map. From the framework of null-hypothesis significant testing, we are unable to interpret the null findings for facets of EI that did not reliably map onto patterns of damage in the current sample. Worth noting, however, is the fact that univariate VLSM is generally unable to detect structure-function relationships that span large swaths of brain damaged in a given sample, as the “control” voxels would be equally associated with the deficit of interest. Given adequate numbers of subjects with overlapping lesion patterns, Multivariate VLSM is an approach intended to increase power to detect the contributions of large regions of brain, in addition to testing the relative necessity of multiple “nodes” significantly associated with some function or deficit (Smith et al., 2013). Finally, the presence of concurrent damage to cortical gray matter and white matter tracts should be taken into account when interpreting the effects of brain lesions; the apparent modularity of some functions in the brain is more fully appreciated in the context of the white matter tracts that connect modules to larger networks. The effect of a focal lesion may arise by impairing the function of individual nodes or the communication between them.

Only when evaluating the neuroimaging literature and loss-of-function findings together, then, does a complete picture of social and emotional cognition emerge. Further research using converging methods is warranted to test whether the mapping of perceiving, using, understanding and managing emotions in the brain can be replicated, both using other brain-lesioned cohorts with impaired EI, and in healthy subjects with naturally distributed ranges of EI.

## Conflict of Interest Statement

The authors declare that the research was conducted in the absence of any commercial or financial relationships that could be construed as a potential conflict of interest.
