# Supplementary material for: Lesion Mapping the Four-Factor Structure of Emotional Intelligence
Source: Front Hum Neurosci. 2015 Dec 10;9:649. doi: 10.3389/fnhum.2015.00649 (PMC4726907; doi:10.3389/fnhum.2015.00649)
Supplement: Supplementary file 1 [file Presentation_1.PDF]

Supplementary Material

Lesion Mapping the Four-Factor Structure of Emotional Intelligence

Joachim T. Operskalski, Erick J. Paul, Roberto Colom, Aron K. Barbey*, and Jordan Grafman

*** Correspondence:** Corresponding Author: barbey@illinois.edu

1 Supplementary Figures 1-5

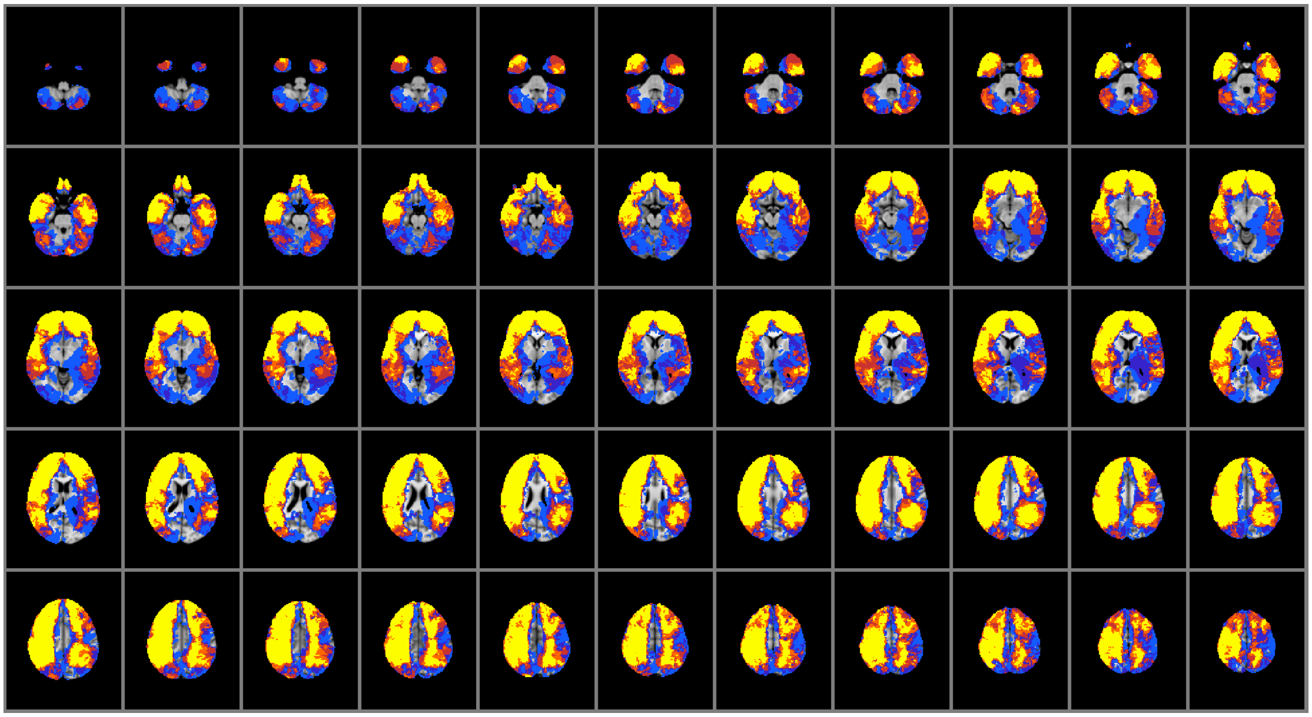

Supplementary Figure 1. Lesion Overlap Map. This heat map displays the overlap of the brain lesions in this sample of injured veterans. Voxels in blue are those with fewer than 3 subjects having sustained damage. Red voxels are those with 3 subjects having sustained damage, orange voxels are those with 4 subjects, and yellow voxels are those with 5 or more subjects. The greatest number of subjects with a lesion in any particular voxel is 21.

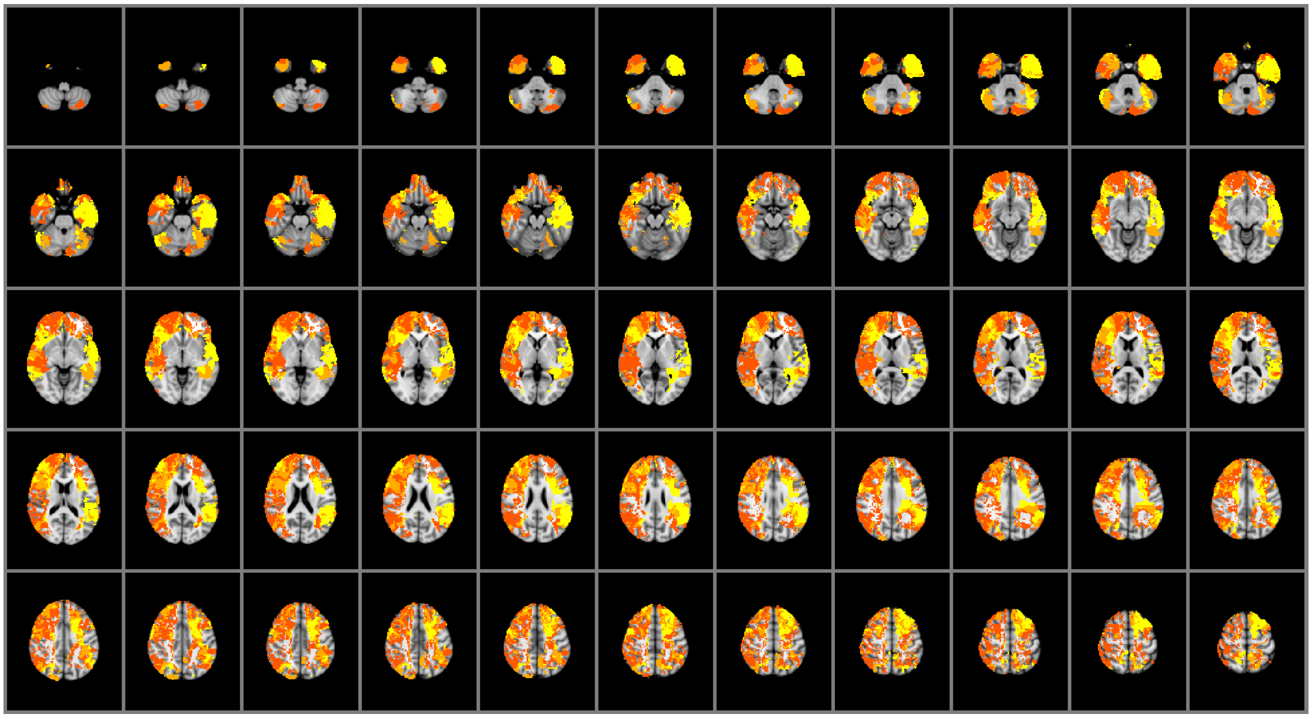

Supplementary Figure 2. Effect size map for perceiving emotions. Standard Cohen's-*d* categories are used to display effect size. Small (dark orange): 0.2-0.49, Medium (light orange): 0.5-0.79, Large (yellow): 0.80-1.0

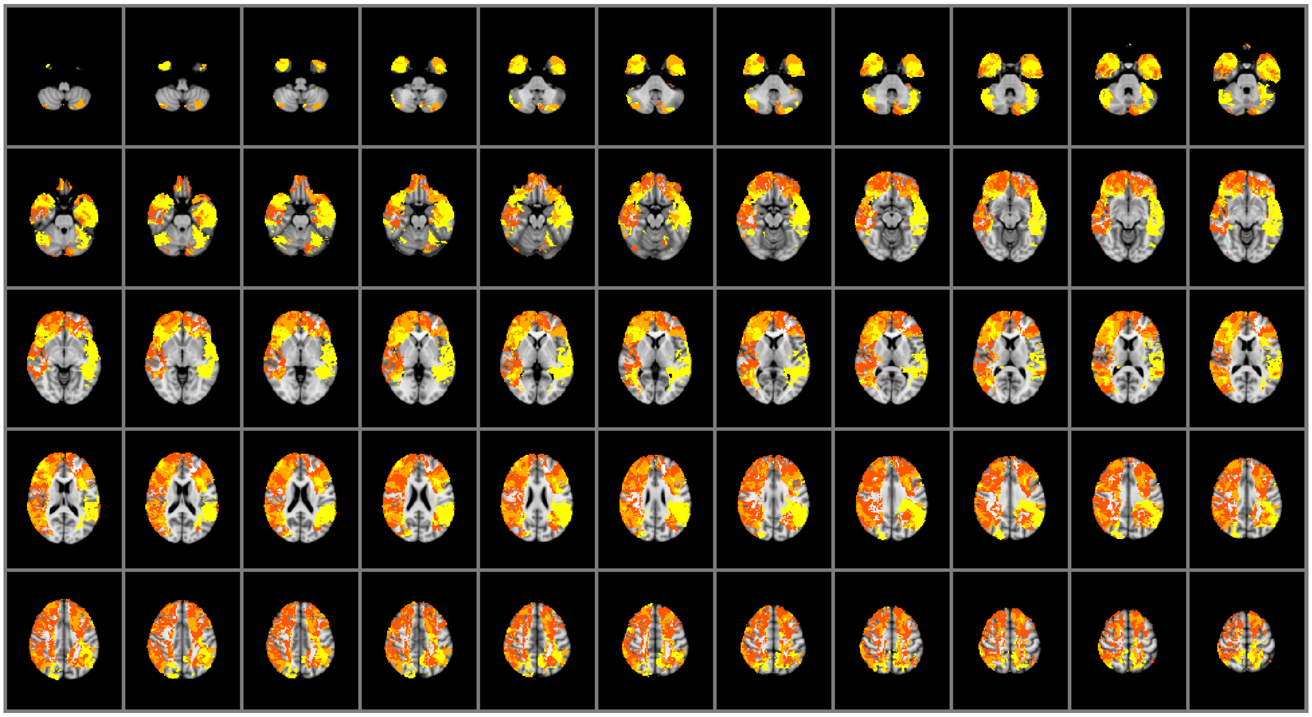

Supplementary Figure 3. Effect size map for managing emotions. Standard Cohen's-*d* categories are used to display effect size. Small (dark orange): 0.2-0.49, Medium (light orange): 0.5-0.79, Large (yellow): 0.80-1.0

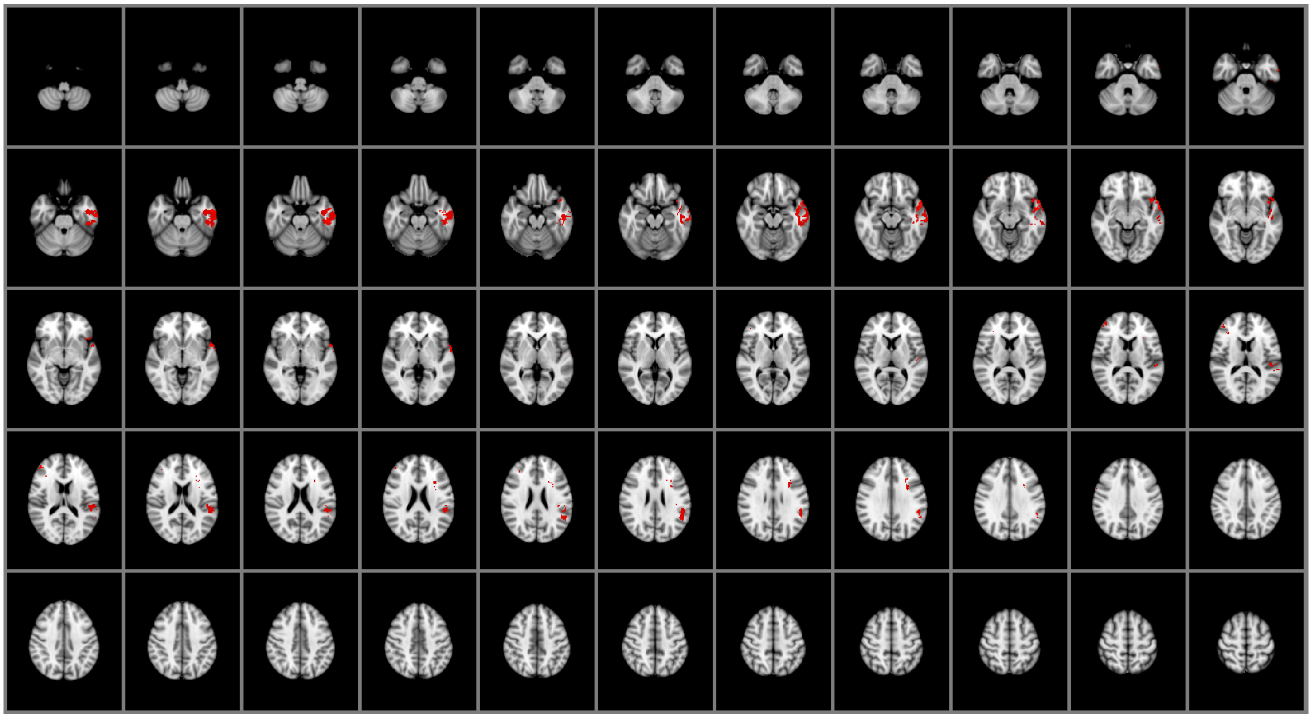

Supplementary Figure 4. VLSM of perceiving emotions – 4 subject minimum. The original analysis was repeated using 4 as the minimum number of subjects with damage in any given voxel for inclusion in the VLSM analysis. Voxels in red are those where damage was significantly associated with impaired ability on the perceiving emotions factor. Structures identified by the 3 subject minimum analysis are all present, but with fewer voxels meeting criterion for significance.

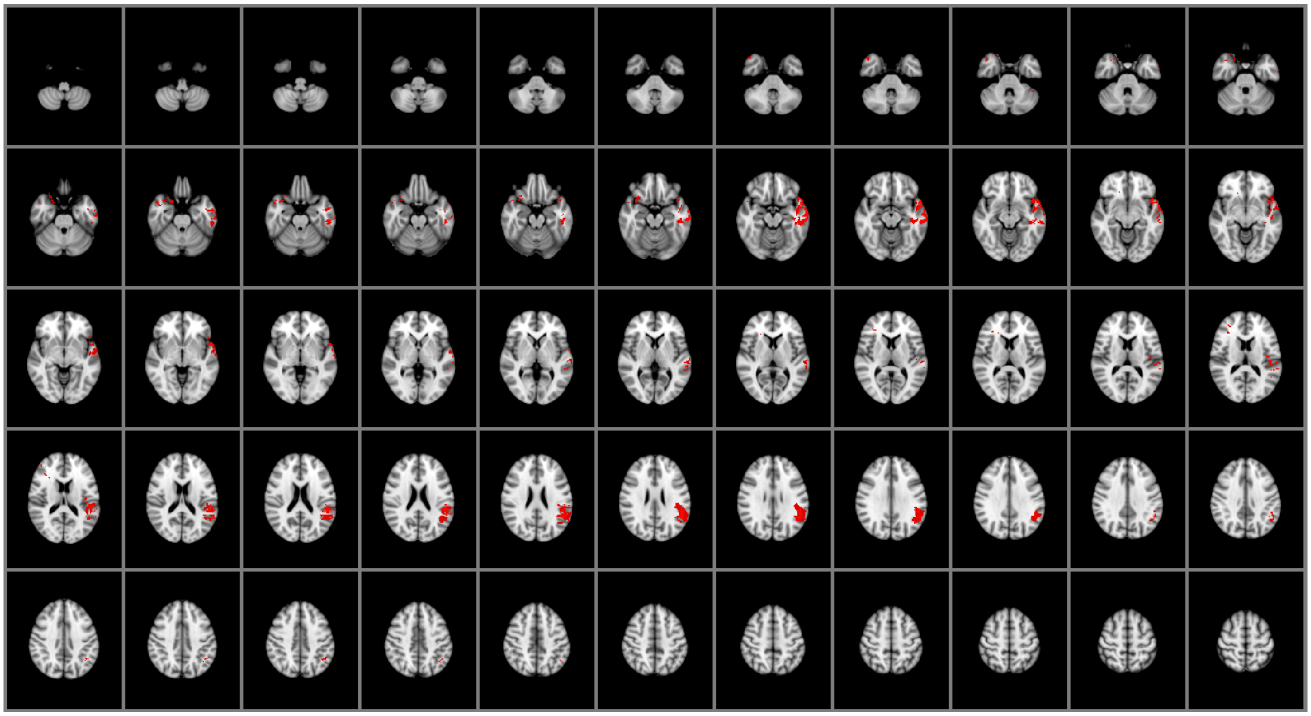

Supplementary Figure 5. VLSM of managing emotions – 4 subject minimum. The original analysis was repeated using 4 as the minimum number of subjects with damage in any given voxel

for inclusion in the VLSM analysis. Voxels in red are those where damage was significantly associated with impaired ability on the managing emotions factor. Structures identified by the 3 subject minimum analysis are all present, but with fewer voxels meeting criterion for significance.

2 Supplementary Tables

	**Perceiving**	**Managing**	**Using**	**Understanding**
**Lesion Size**	-.24*	-.24*	-.13	-.28*

Supplementary Table 1. Correlation with lesion size. Impairment in emotional intelligence is correlated with lesion size. *: ($p < 0.05$).
